# Determination and diagnostic value of *CA9* mRNA in peripheral blood of patients with oral leukoplakia

**DOI:** 10.1080/14756366.2018.1466120

**Published:** 2018-05-10

**Authors:** Manuel Torres López, Mario Pérez Sayáns, Cintia Chamorro Petronacci, Francisco Barros Angueira, Pilar Gándara Vila, Alejandro Lorenzo Pouso, Abel García García

**Affiliations:** a Oral Medicine, Oral Surgery and Implantology Unit, Faculty of Medicine and Dentistry, Instituto de Investigación Sanitaria de Santiago (IDIS), Santiago de Compostela, Spain;; b Unidad de Medicina Molecular – Fundación Pública Galega de Medicina Xenómica, Edificio de Consultas planta 2, Hospital Clinico Universitario, Santiago de Compostela, Spain

**Keywords:** CA9**;** oral squamous cell carcinoma**;** leukoplakia

## Abstract

**Background:** Oral leukoplakia is one of the most common oral premalignant disorder. The classical evaluation through tissue biopsy is not always valid to evaluate the risk of malignization.

**Material and methods:** RT-qPCR was performed on 47 blood samples (21 patients with leukoplakia, 2 with oral squamous cell carcinoma (OSCC), and 24 healthy patients) and on 11 tissue samples (3 leukoplakia, 4 OSCC, and 4 samples of healthy tissue).

**Results:** There are significant differences in expression between the different groups (*F* = 4.057, *p* = .006). The Duncan *post hoc* test shows that the only group that differentiates is the tumour tissue. Using Wilcoxon test, different covariables of patients with leukoplakia were analysed with respect to the group of healthy patients and no significant differences were observed.

**Conclusions:** The diagnostic route through liquid biopsy has not been conclusive in this study, but there are significant differences in the levels analysed in the different tissue samples.

## Introduction

Among the tumours that most frequently affect the oral cavity, oral squamous cell carcinoma (OSCC) represents 90% of malignant oral neoplasms^1^. OSCC is one of the most heterogeneous diseases, with a multifactorial aetiology, among which tobacco and alcohol stand out as risk factors. Due to a generally late diagnosis, the 5-year advanced OSCC survival rate is less than 9%^2^; therefore, an early diagnosis is fundamental for patient management and prognosis.

**Figure 1.**
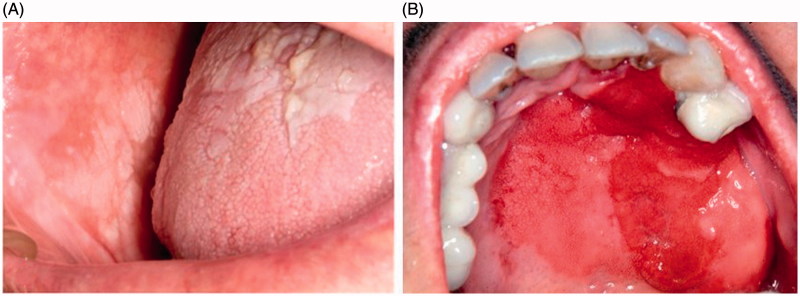
(A) Non-homogeneous leukoplakia in buccal mucosa and tongue. (B) Etrytroplasia in hard palate.

Oral leukoplakia is the disorder with the highest malignant potential of the mucosa of the oral cavity and can result in OSCC^3^, with a current prevalence of approximately 1–2% in all ages^4^ (Figure 1). Currently, the only marker of malignancy recognised by most authors is the degree of dysplasia^5–7^, but this type of assessment is very subjective, with great observational variability, as there are still no quantifiable parameters.

At present, biopsy and histopathological analysis remain the "*gold standard*" of diagnostic tests. Due to the great potential for malignant transformation of oral leukoplakia, it is essential to search for prognostic biomarkers, objectively agreeing on the results, to help identify the evolution of the lesions and to help the clinician to better manage them. It is also necessary to evolve towards a less invasive method to conduct these studies. Liquid biopsy is a non-invasive tool for the discovery of biomarkers that is generating great expectations. Liquid biopsies (in blood, urine, saliva, etc.), due to their minimally invasive nature, are associated with significantly lower morbidity and can be performed more frequently to obtain a better follow-up of the disease^8^.

Carbonic anhydrases are a broad group of metalloenzymes involved in numerous physiological and pathological processes. Specifically, carbonic anhydrase IX is found in hypoxic tumour tissues of different types of cancer. According to several studies, CA9 increases its expression with the increase in tumour stage, likely related to the degree of hypoxia, and could be used as a prognostic factor^9–13^.

The present work addresses the determination of CA9 in oral leukoplakia by quantitative PCR in blood and tissues as a diagnostic and prognostic method for better management of patients with said disease.

## Materials and methods

### Study samples

The study protocol was approved by the regional ethics committee of Galicia (comité de ética autonómico de Galicia, CAEI), reference 2016/337. The informed consent of participation for each patient was obtained, and peripheral blood samples from 47 patients and 11 tissue samples were collected, both from patients with leukoplakia and from healthy donors treated in the Oral Medicine Service of the Faculty of Dentistry from the University of Santiago de Compostela, between 2014 and 2016. All patients were diagnosed clinically and histologically prior to taking samples.

The following were the inclusion and exclusion criteria:

Inclusion: patients with leukoplakia-like clinical lesions that were subsequently confirmed by biopsy and from which clinical records and photographs are available.

Exclusion:

1. Patients with the clinical appearance or histological findings of white lesions, such as leukoedema, linea alba, lesions such as lichen planus, lupus erythematosus, etc.
2. Patients with follow-up periods of less than 1 year.
3. Patients for whom there are no clinical images of the lesions to be studied.
4. Patients without initial histological diagnosis.
5. Patients whose anatomopathological materials are not available to confirm the initial diagnosis.

### Blood samples

Of the 47 blood samples available to us, 21 correspond to patients who were diagnosed with leukoplakia, and 2 were from OSCC patients. Nine of the study patients were men (39.1%), and 14 were women (60.9%), with an age range between 51 and 89 years (mean: 70.21, standard deviation (SD): 9.82). The other 24 samples came from healthy donors with no medical history of interest, including 10 men (41.7%) and 14 women (58.3%), with an age range between 21 and 65 years (mean: 33.83, SD: 13.99). The details of the patients are described in Table 1. Blood extraction was performed using a disposable method; blood was collected in a tube containing the anticoagulant EDTA-K_3_ (BD Vacutainer^®^ K3E 7.2 mg, ref 368860, 4.0 ml). The location of the venous puncture was cleaned with chlorhexidine, an antiseptic with bactericidal and fungicidal functions.

**Table 1.** Characteristics of the study sample.

Patients	Blood case	Tissue case	Blood control	Tissue control
Sex				
Male	9 (39.1)	3 (42.9)	10 (41.7)	3 (75)
Female	14 (60.9)	4 (57.1)	14 (58.3)	1 (25)
Smoker				
Yes	4 (17.4)	2 (28.6)	5 (20.8)	1 (25)
No	19 (82.6)	5 (71.4)	19 (79.2)	3 (75)
Mean age	70.21 (±9.23)	62.85 (±6.46)	33.83 (±13.99)	48.25 (±23.62)
Lesion localisation				
Tongue	8 (34.8)	1 (14.2)		
Gum	3 (13)	3 (42.8)		
Buccal mucosa	2 (8.7)	–		
Mouth floor	–	1 (14.3)		
Multiple	10 (43.5)	2 (28.6)		
Leukoplakia analysis				
Nodular	9 (39.1)	1 (33.3)		
Verrucous	14 (60.9)	2 (66.6)		
T stage				
T1–T2	3 (100)	3 (75)		
T3–T4	–	1 (25)		
Recurrence^a^				
Yes	19 (95)	4 (57.1)		
No	1 (5)	3 (42.9)		

Values are presented as *N* (%).

^a^Decreased number of patients due to death.

### Tissue samples

We also obtained 11 tissue samples; 4 samples belonged to the oral mucosa of healthy patients, and 3 corresponded to leukoplakias and 4 to carcinomas (all samples were diagnosed histologically), of which 6 were from men (54.5%) and 5 were from women (45.5%), with an age range of between 15 and 76 years (mean: 57.54; DS: 3.51). The samples were obtained by performing a circumferential anaesthetic block to the lesion and removing the most representative area of the lesion using a cold scalpel. Immediately after taking the sample, it was immersed in liquid nitrogen (−180 °C) to avoid RNA degradation and was subsequently stored at −80 °C.

### RNA isolation and cDNA synthesis

The total RNA from the blood samples was extracted using a Maxwell RSC simplyRNA Blood Purification kit following the manufacturer's instructions (Promega, Madison, WI). Frozen tissues were homogenised with chilled 1-thyoglycerol/homogenization solution, and total RNA was extracted using a Maxwell 16 LEV tissue kit simplyRNA following the manufacturer's instructions (Promega, Madison, WI). RNA concentration and quality were determined in a NanoDrop ND1000 spectrophotometer (Thermo Fisher Scientific, Wilmington, DE).

Five hundred nanograms of total RNA, obtained from the blood and tissue samples, was used in the cDNA synthesis reactions performed with random hexamer primers (Thermo Fisher Scientific, Wilmington, DE), SuperScript II Reverse Transcriptase (Invitrogen, Carlsbad, CA) and an RNA ribonuclease inhibitor (Promega, Madison, WI). The conditions of the reverse transcription-polymerase chain reaction (RT-PCR) were 20 °C for 10 min followed by 50 min at 42 °C, and the enzyme was inactivated at 95 °C for 3 min.

### Real-time quantitative PCR

For the quantitative real-time PCR studies, a *CA9* gene-specific probe Hs00154208_m1 was used for TaqMan Gene Expression Assays (Thermo Fisher Scientific, Wilmington, DE), and a specific probe for the *ABL* gene from the Ipsogen BCR-ABL1 Mbcr IS-MMR Kit (Qiagen, Venlo, ND) was used as a control or normalising gene. PCR samples were prepared using a KapaFast commercial ABI PRISM qPCR kit (KapaBiosystems, Wilmington, MA). The samples were analysed in a StepOne Plus Real Time PCR system (Thermo Fisher Scientific, Wilmington, DE) following the manufacturer's instructions. The reaction conditions were as follows: 95 °C for 10 min followed by 40 cycles of 15 s at 95 °C and 1 min at 60 °C. Each sample was made in duplicate. As a positive RT-PCR control, cDNA extracted from HeLa cells was used^14^. Positive controls in duplicate and negative controls with water instead of nucleic acid were included on each experimental plate. The baseline and threshold were automatically adjusted, and Ct values were determined for each sample using StepOne v2.3 software.

To normalise the expression values, the ΔCt, ΔCt = Ct *AB*L-Ct *CA9 method was* used, with *ABL* as the reference gene. The relative concentration of *CA9* was determined using the 2^−ΔCt^ method. The data were processed using Data Assist v3.0.1 (Thermo Fisher Scientific, Wilmington, DE). The following parameters were used:

Maximum allowed Ct value: 40.0

Include maximum values of Ct in calculations: yes

Exclude *outliers* between replicates: yes

Adjust *p* values using the False Discovery Rate of Benjamini–Hochberg: yes

Standardization method: endogenous control.

### Statistical analysis

The software analyses the differences of the relative values of *CA9* expression between each group (blood samples from cases, tissue samples from cases and controls and tumours) and the reference group (blood controls/healthy) using a Wilcoxon test, assuming that the variances between the groups are not homogeneous. This same analysis has been used to explore the possible existence of differentiation between blood samples (controls and cases), stratifying the cases according to various clinical variables.

Complimentarily, the differentiation in expression between the 5 groups was analysed by analysis of variance (ANOVA); because an analysis of variance can show significant results without implying that all of the analysed groups are significantly different from each other, a Duncan *post hoc* test was performed, which evaluates the significance in each pair of combinations and allows the differentiation of which group or groups differ from the rest.

All statistical analyses were performed using IBM SPSS Statistics software version 20.0.

## Results

### Determination of the expression level of CA9

To determine the level of expression of the *CA9* gene, the comparative relative quantification method described by Livak et al.^15^, known as the 2^−ΔCt^ method, was used.

A total of 54 of the 58 selected samples were analysed; 4 were discarded for not meeting the established quality requirements. The *CA9* values were normalised using the *ABL* gene as the control gene by the ΔCt method. As established by the technique, the Ct values obtained for the *ABL* gene were lower than those of the *CA9* gene. In 3 samples, it was not possible to determine the Ct value for *CA9*, and the software automatically assigned the Ct value as 40, as the minimum value admitted. Therefore, in these samples, the presence of *CA9* was not detected; however, *ABL* was detected, which verified the presence of cDNA. A single sample from the blood of a healthy patient was excluded from the analyses because no signal was obtained for either of the 2 genes. The Ct values obtained are presented in Annex I.

The presence of *CA9* was detected in all samples from tumour tissues and leukoplakias and in both the HeLa positive control included in the study and the patient samples. Tissue samples from healthy patients had low expression levels for *CA9*. Among the blood samples, the Ct values for *CA9* obtained were in the single-copy range (Ct 34–40), which could be considered as *background noise,* and *CA9* was not detected in 3 samples: 2 healthy donors and 1 patient with leukoplakia. The Ct values for these same samples for *ABL* ranged from 25 to 33.

For a patient with leukoplakia, a tissue sample and a blood sample were available; in this case, the expression of *CA9* was detected in the tissue sample with leukoplakia but not in the blood samples. The follow-up performed allowed us to determine that this case later became malignant with the development of a OSCC; however, the *CA9* expression level was not higher than in the other leukoplakias.

From the Ct values obtained for both genes, the 2^−ΔCt^ method was normalised and applied, using the group of control blood samples as a calibrator, to obtain values for relative quantification.

### Statistical analysis of the CA9 expression level

Table 2 shows the descriptive values (mean and standard error) for 2^−ΔCt^ in each of the 5 groups and the results of their comparison with the control blood samples: RQ (*fold change*) – obtained from dividing each average value between the mean value of the reference group (blood of healthy patients) – and the probability of the Wilcoxon test with which their differentiation was analysed.

**Table 2.** Descriptive statistics for 2^−ΔCt^ and results of comparative analysis.

	2^−ΔCT^			Comparison vs healthy patient blood		ANOVA (5 groups)
Group	*N*	Mean	st error	FC (RQ)	Prob	DUNCAN
Healthy patient blood	23	0.0049	0.0012	1.0000	***–***	A
Leukoplakia patient blood	22	0.0037	0.0010	0.7473	0.4117	A
Healthy tissue	3	0.0189	0.0066	3.8325	0.0283	A
Leukoplakia tissue	2	0.0848	0.0126	17.1500	0.0336	A
Tumor tissue	4	6.0330	5.8164	1220.4994	0.0054	B

Additionally, and globally, we analysed the existence of significant differences in 2^−ΔCt^ between the 5 groups of samples analysed – tumour tissue, tissue with leukoplakia, healthy tissue, blood from patients with leukoplakia, and blood from healthy donors – by analysis of variance. The results were clearly significant (*F* = 4.057, *p* = 0.006), indicating that there were significant differences in expression between the groups. However, the Duncan *post hoc* test (Table 2) showed that the only group that differentiated was the tumour tissue; all of the others showed non-significant differences among themselves; therefore, they formed a single group. These results do not vary if sample 51, a sample of stage 3 tumour tissue with extremely high expression values, is excluded from the analysis; however, in this case, the RQ value for tumour tissue would decrease to 44.06.

If the ΔCt values are used as an expression measure (Table 3), ANOVA again indicated significant differences (*F* = 19.65, *p* < 0.0001), but the differentiation of the groups showed a more gradual pattern: the tumour tissue did not differentiate from the leukoplastic tissue, which in turn could not be differentiated from the tissue of healthy patients, again emphasising the lack of discrimination between the blood samples of subjects with leukoplakia and healthy controls.

**Table 3.** ANOVA analysis and DUNCAN test for ΔCt.

	deltaCt			ANOVA (5 groups)
Group	*N*	mean	st error	DUNCAN
Healthy patient blood	22	8.4308	0.3324	A
Leukoplakia patient blood	23	8.7383	0.2787	A
Healthy tissue	3	5.9075	0.5248	B
Leukoplakia tissue	2	3.5763	0.2155	BC
Tumor tissue	4	1.3411	2.1780	C

Additionally, and in an exploratory way, to evaluate the possible existence of differences between subgroups of the blood samples of the subjects with leukoplakia and the controls in detail, the Wilcoxon test was performed, stratifying the patients with leukoplakia according to the covariables of interest (clinical type of lesion, maximum degree of dysplasia and location), comparing them in each sub-analysis with the controls. Again, the results were not significant (Table 4).

**Table 4.** Mean 2^−ΔCt^ values, fold change and Wilcoxon test probability results for each group.

	Mean value		No. patients			Wilcoxon test
Analysis stratified according to	Patient blood leukoplakia	Patient blood healthy	With leukoplakia	Healthy	FC	*p*
Clinical type						
Nodular leukoplakia	0.0068	0.0049	7	23	1.37	0.8081
Verrucous leukoplakia	0.0022	0.0049	14	23	0.45	0.2241
Localization						
Tongue leukoplakia	0.0036	0.0049	8	23	0.72	0.4102
Multiple lesion leukoplakia	0.0026	0.0049	10	23	0.53	0.4279
Dysplasia location						
Leukoplakia with dysplasia in the tongue	0.0039	0.0049	7	23	0.84	0.4357
Leukoplakia with dysplasia in the buccal mucosa	0.0026	0.0049	4	23		
Maximum degree of dysplasia					0.79	0.4979
Leukoplakia without dysplasia	0.0042	0.0049	8	23	0.52	0.8131
Leukoplakia with slight dysplasia	0.0025	0.0049	4	23	0.84	0.4357
Leukoplakia with moderate dysplasia	0.0054	0.0049	5	23	0.50	0.6130
Leukoplakia with severe dysplasia	0.0022	0.0049	4	23	1.09	0.7665

## Discussion

The objective of this study was to evaluate the possibility of detecting the level of CA9 expression in the blood of patients with leukoplakia by means of quantitative real-time PCR for use as a biomarker. In a previous study, we observed that CA9 can be detected by immunohistochemistry in these lesions and, according to our series of cases, the absence of CA9 could exclude the possibility of dysplasia. Therefore, the detection of CA9 in blood, without the need to perform a biopsy of the affected tissue, could be useful during patient follow-up.

To this end, a group of patients with leukoplakia, confirmed by biopsy, and a group of healthy donors were selected to determine if there are differences in *CA9* gene expression levels between both groups. The main difficulty of this study involved collecting a large number of patients with oral leukoplakia who had been previously diagnosed histopathologically, which limits its statistical power. OSCC and leukoplakia tissue samples and healthy tissue samples were selected to determine the *CA9* expression levels in these circumstances. As already mentioned, the presence of CA9 has been widely described in different tumours; however, the protein is not expressed only in normal tissues. As a positive control for the reaction, cDNA obtained from HeLa cells was included, in which the Pastoreka group identified the protein in 1992; therefore, we know that the cells express the protein.

The results were positive for the OSCC and leukoplakia samples. The highest *CA9* gene expression value was found in a stage T3 OSCC sample. In previous studies, we had already observed, using other technologies, that CA9 is overexpressed in OSCC tumour tissues, with statistically significantly higher expression levels in advanced stages of the tumour^16^^,^^17^. The presence of CA9 in these samples coincides with these previous findings. The ΔCt values in the OSCC and leukoplakia tissue samples differed significantly from the tissues samples from healthy patients and from blood samples from both healthy patients and those with leukoplakia.

However, between the two groups of blood samples analysed from healthy patients and patients with leukoplakia, the expression levels were minimal, and there were no statistically significant differences in the expression between these two groups.

The detection of CA9 in blood and other tissues using this method has previously been successful^18^. In the study by Takacova et al. in renal cell carcinomas, 74 samples of blood and tissue from patients were analysed, and *CA9* expression was detected in 24 of them, with a specificity of 100%, with no CA9-positive sample among the control patients. The authors found that the *CA9* expression was greater in the initial stages of the tumour. De Martina et al. analysed the presence of urinary CA9 as a diagnostic marker in bladder urothelial cancer using the same method. CA9 was detected in 169 of the 196 patients analysed and in 6 of 123 controls, with a sensitivity of 86.2% and a specificity of 95.1%. The authors obtained higher levels of CA9 in tumours at lower stages compared with the more advanced stages^9^. In these cases, it is necessary to consider that these were patients with tumours and the presence of CA9 in CTCs (circulating tumour cells) was being identified in blood, as the dispersion of neoplastic cells from the primary tumour is a crucial point of the metastasis process.

There are 2 isoforms for the *CA9* gene (1 complete, FL, and another in which exons 8 and 9, AS, are lost) that can be detected in normal tissues and independently of the level of hypoxia. Thus, the probe design is critical for detecting the complete isoform; we took this point into account in the design of the experiment and opted for a commercial probe^19^. The detection of *CA9* in tumour samples, the positive HeLa control and leukoplakias confirmed the efficacy of the method used; therefore, the *CA9* expression levels in the blood of patients with leukoplakia were very low and did not differ from those found in healthy tissues and blood controls.

Although the determination of a diagnostic and follow-up pathway for premalignant lesions based on liquid biopsy and CA9 detection using this technique has not been possible, it is necessary to continue investigating this protein and its relationship with different pathologies, recognising in which fluids it is expressed, for a more accurate study for each type of injury.

## Supplementary Material

IENZ_1466120_Supplementary_Material.pdf
