# Supplementary material for: Determination and diagnostic value of CA9 mRNA in peripheral blood of patients with oral leukoplakia
Source: J Enzyme Inhib Med Chem. 2018 May 10;33(1):951–5. doi: 10.1080/14756366.2018.1466120 (PMC6009864; doi:10.1080/14756366.2018.1466120)
Supplement: IENZ_1466120_Supplementary_Material.pdf [file IENZ_A_1466120_SM3815.pdf]

ºTabla con valores de Ct

Patient	Sample Group	Genee	Ct	Ct adjusted
Control hela	HELA	ABL	23,5814	23,5814
Control hela	HELA	ABL	23,2884	23,2884
Control hela	HELA	ABL	23,3427	23,3427
Control hela	HELA	ABL	23,4132	23,4132
Control hela	HELA	ABL	23,2377	23,2377
Control hela	HELA	ABL	23,1367	23,1367
Control hela	HELA	CA9	25,2953	25,2953
Control hela	HELA	CA9	25,2644	25,2644
Control hela	HELA	CA9	25,4917	25,4917
Control hela	HELA	CA9	25,2033	25,2033
Control hela	HELA	CA9	25,9093	25,9093
Control hela	HELA	CA9	25,7896	25,7896
1	Blood leukoplakia sample	ABL	27,073	27,073
1	Blood leukoplakia sample	ABL	27,0361	27,0361
1	Blood leukoplakia sample	CA9	36,5402	36,5402
1	Blood leukoplakia sample	CA9	36,1042	36,1042
2	Blood leukoplakia sample	ABL	26,9159	26,9159
2	Blood leukoplakia sample	ABL	26,8047	26,8047
2	Blood leukoplakia sample	CA9	34,6513	34,6513
2	Blood leukoplakia sample	CA9	34,3632	34,3632
3	Blood leukoplakia sample	ABL	26,4249	26,4249
3	Blood leukoplakia sample	ABL	26,305	26,305
3	Blood leukoplakia sample	CA9	34,8295	34,8295
**Patient**	**Sample Group**	**Gene**	**Ct**	**Ct adjusted**

3	Blood leukoplakia sample	CA9	36,7868	36,7868
4	Blood leukoplakia sample	ABL	26,5187	26,5187
4	Blood leukoplakia sample	ABL	26,5313	26,5313
4	Blood leukoplakia sample	CA9	35,1701	35,1701
4	Blood leukoplakia sample	CA9	34,2211	34,2211
5	Blood leukoplakia sample	ABL	27,3896	27,3896
5	Blood leukoplakia sample	ABL	27,5234	27,5234
5	Blood leukoplakia sample	CA9	35,723	35,723
6	Blood leukoplakia sample	ABL	28,4569	28,4569
6	Blood leukoplakia sample	ABL	28,4203	28,4203
6	Blood leukoplakia sample	CA9	37,2957	37,2957
6	Blood leukoplakia sample	CA9	38,0139	38,0139
7	Blood leukoplakia sample	ABL	27,1688	27,1688
7	Blood leukoplakia sample	ABL	27,1755	27,1755
7	Blood leukoplakia sample	CA9	35,7265	35,7265
7	Blood leukoplakia sample	CA9	34,7815	34,7815
8	Blood leukoplakia sample	ABL	26,7204	26,7204
8	Blood leukoplakia sample	ABL	26,8416	26,8416
**Patient**	**Sample Group**	**Gene**	**Ct**	**Ct adjusted**

8	Blood leukoplakia sample	CA9	35,5768	35,5768
8	Blood leukoplakia sample	CA9	34,5944	34,5944
8	Blood leukoplakia sample	CA9	34,993	34,993
8	Blood leukoplakia sample	CA9	36,3709	36,3709
9	Blood leukoplakia sample	ABL	26,9734	26,9734
9	Blood leukoplakia sample	ABL	26,8902	26,8902
9	Blood leukoplakia sample	CA9	35,1623	35,1623
10	Blood leukoplakia sample	ABL	30,9742	30,9742
10	Blood leukoplakia sample	ABL	30,9153	30,9153
10	Blood leukoplakia sample	CA9	36,8946	36,8946
11	Blood leukoplakia sample	ABL	28,6297	28,6297
11	Blood leukoplakia sample	CA9	36,5868	36,5868
11	Blood leukoplakia sample	CA9	36,3849	36,3849
12	Blood leukoplakia sample	ABL	25,9463	25,9463
12	Blood leukoplakia sample	ABL	25,8385	25,8385
12	Blood leukoplakia sample	CA9	37,0579	37,0579
12	Blood leukoplakia sample	CA9	36,3197	36,3197
12	Blood leukoplakia sample	CA9	37,7687	37,7687
**Patient**	**Sample Group**	**Gene**	**Ct**	**Ct adjusted**

12	Blood leukoplakia sample	CA9	36,2126	36,2126
13	Blood leukoplakia sample	ABL	26,9089	26,9089
13	Blood leukoplakia sample	ABL	25,1044	25,1044
13	Blood leukoplakia sample	CA9	34,1031	34,1031
13	Blood leukoplakia sample	CA9	34,7411	34,7411
14	Blood leukoplakia sample	ABL	28,045	28,045
14	Blood leukoplakia sample	ABL	26,0586	26,0586
14	Blood leukoplakia sample	CA9	36,6717	36,6717
14	Blood leukoplakia sample	CA9	35,3739	35,3739
15	Blood leukoplakia sample	ABL	28,8879	28,8879
15	Blood leukoplakia sample	ABL	28,7928	28,7928
15	Blood leukoplakia sample	CA9	37,1521	37,1521
15	Blood leukoplakia sample	CA9	undetermined	40
16	Blood leukoplakia sample	ABL	31,3117	31,3117
16	Blood leukoplakia sample	ABL	31,3387	31,3387
16	Blood leukoplakia sample	CA9	undetermined	40
16	Blood leukoplakia sample	CA9	undetermined	40
17	Blood leukoplakia sample	ABL	32,0522	32,0522
**Patient**	**Sample Group**	**Gene**	**Ct**	**Ct adjusted**

17	Blood leukoplakia sample	ABL	321.238	32,1238
17	Blood leukoplakia sample	CA9	37.785	37,785
18	Blood leukoplakia sample	ABL	253.056	25,3056
18	Blood leukoplakia sample	ABL	252.641	25,2641
18	Blood leukoplakia sample	CA9	345.337	34,5337
18	Blood leukoplakia sample	CA9	349.138	34,9138
18	Blood leukoplakia sample	ABL	264.178	26,4178
18	Blood leukoplakia sample	ABL	264.722	26,4722
18	Blood leukoplakia sample	CA9	348.481	34,8481
18	Blood leukoplakia sample	CA9	352.197	35,2197
19	Blood leukoplakia sample	ABL	273.356	27,3356
19	Blood leukoplakia sample	ABL	273.905	27,3905
19	Blood leukoplakia sample	CA9	undetermined	40
19	Blood leukoplakia sample	CA9	35,8366	35,8366
20	Blood leukoplakia sample	ABL	28,3	28,3
20	Blood leukoplakia sample	ABL	28,328	28,328
20	Blood leukoplakia sample	CA9	undetermined	40
20	Blood leukoplakia sample	CA9	36,399	36,399
**Patient**	**Sample Group**	**Gene**	**Ct**	**Ct adjusted**

21	Blood leukoplakia sample	ABL	25,854	25,854
21	Blood leukoplakia sample	ABL	25,7852	25,7852
21	Blood leukoplakia sample	CA9	37,4851	37,4851
21	Blood leukoplakia sample	CA9	35,3558	35,3558
22	Blood healthy patient	ABL	31,0935	31,0935
22	Blood healthy patient	ABL	31,2869	31,2869
22	Blood healthy patient	CA9	37,0302	37,0302
22	Blood healthy patient	CA9	37,7119	37,7119
23	Blood healthy patient	ABL	31,8735	31,8735
23	Blood healthy patient	ABL	31,9638	31,9638
23	Blood healthy patient	CA9	38,2822	38,2822
23	Blood healthy patient	CA9	undetermined	40
24	Blood healthy patient	ABL	30,5648	30,5648
24	Blood healthy patient	ABL	30,2583	30,2583
24	Blood healthy patient	CA9	undetermined	40
24	Blood healthy patient	CA9	37,4665	37,4665
25	Blood healthy patient	ABL	32,2128	32,2128
25	Blood healthy patient	ABL	32,0952	32,0952
25	Blood healthy patient	CA9	37,9358	37,9358
25	Blood healthy patient	CA9	36,9853	36,9853
26	Blood healthy patient	ABL	28,0264	28,0264
26	Blood healthy patient	ABL	28,1654	28,1654
26	Blood healthy patient	CA9	36,2473	36,2473
26	Blood healthy patient	CA9	36,4254	36,4254
**Patient**	**Sample Group**	**Gene**	**Ct**	**Ct adjusted**
27	Blood healthy patient	ABL	26,9817	26,9817

27	Blood healthy patient	ABL	26,7214	26,7214
27	Blood healthy patient	CA9	34,737	34,737
27	Blood healthy patient	CA9	34,9976	34,9976
28	Blood healthy patient	ABL	33,3446	33,3446
28	Blood healthy patient	ABL	33,0539	33,0539
28	Blood healthy patient	CA9	undetermined	40
28	Blood healthy patient	CA9	undetermined	40
29	Blood healthy patient	ABL	32,14	32,14
29	Blood healthy patient	ABL	32,1451	32,1451
29	Blood healthy patient	CA9	undetermined	40
29	Blood healthy patient	CA9	38,1734	38,1734
30	Blood healthy patient	ABL	25,5421	25,5421
30	Blood healthy patient	ABL	25,4532	25,4532
30	Blood healthy patient	CA9	34,6065	34,6065
30	Blood healthy patient	CA9	35,4536	35,4536
31	Blood healthy patient	ABL	25,4662	25,4662
31	Blood healthy patient	ABL	25,5529	25,5529
31	Blood healthy patient	CA9	34,6645	34,6645
31	Blood healthy patient	CA9	36,1388	36,1388
32	Blood healthy patient	ABL	27,3468	27,3468
32	Blood healthy patient	ABL	27,379	27,379
32	Blood healthy patient	CA9	35,6702	35,6702
33	Blood healthy patient	ABL	30,3866	30,3866
33	Blood healthy patient	ABL	30,476	30,476
33	Blood healthy patient	CA9	37,9632	37,9632
**Patient**	**Sample Group**	**Gene**	**Ct**	**Ct adjusted**
34	Blood healthy patient	ABL	31,1863	31,1863
34	Blood healthy patient	CA9	36,9566	36,9566

34	Blood healthy patient	CA9	38,5731	38,5731
35	Blood healthy patient	ABL	31,0089	31,0089
35	Blood healthy patient	ABL	31,048	31,048
35	Blood healthy patient	CA9	undetermined	40
35	Blood healthy patient	CA9	undetermined	40
36	Blood healthy patient	ABL	26,0034	26,0034
36	Blood healthy patient	ABL	26,0221	26,0221
36	Blood healthy patient	CA9	35,7907	35,7907
36	Blood healthy patient	CA9	36,5358	36,5358
37	Blood healthy patient	ABL	27,6305	27,6305
37	Blood healthy patient	ABL	26,4948	26,4948
37	Blood healthy patient	CA9	37,6575	37,6575
37	Blood healthy patient	CA9	34,992	34,992
38	Blood healthy patient	ABL	26,914	26,914
38	Blood healthy patient	ABL	26,863	26,863
38	Blood healthy patient	CA9	35,445	35,445
38	Blood healthy patient	CA9	34,803	34,803
39	Blood healthy patient	ABL	27,0263	27,0263
39	Blood healthy patient	ABL	27,1306	27,1306
39	Blood healthy patient	CA9	35,5105	35,5105
39	Blood healthy patient	CA9	33,9456	33,9456
40	Blood healthy patient	ABL	28,5257	28,5257
40	Blood healthy patient	ABL	28,2063	28,2063
40	Blood healthy patient	CA9	undetermined	40
**Patient**	**Sample Group**	**Gene**	**Ct**	**Ct adjusted**
40	Blood healthy patient	CA9	36,3744	36,3744
41	Blood healthy patient	ABL	26,0122	26,0122
41	Blood healthy patient	ABL	26,1119	26,1119

41	Blood healthy patient	CA9	36,1497	36,1497
41	Blood healthy patient	CA9	37,6402	37,6402
42	Blood healthy patient	ABL	27,8563	27,8563
42	Blood healthy patient	ABL	27,748	27,748
42	Blood healthy patient	CA9	36,3522	36,3522
42	Blood healthy patient	CA9	35,588	35,588
43	Blood healthy patient	ABL	27,3914	27,3914
43	Blood healthy patient	ABL	27,2777	27,2777
43	Blood healthy patient	CA9	35,7572	35,7572
43	Blood healthy patient	CA9	39,0368	39,0368
43	Blood healthy patient	ABL	25,9869	25,9869
44	Blood healthy patient	ABL	25,9725	25,9725
44	Blood healthy patient	CA9	undetermined	40
44	Blood healthy patient	CA9	35,7205	35,7205
45	Healthy tissue	ABL	28,0829	28,0829
45	Healthy tissue	ABL	28,1278	28,1278
45	Healthy tissue	CA9	34,8158	34,8158
45	Healthy tissue	CA9	35,0194	35,0194
46	Healthy tissue	ABL	26,8332	26,8332
46	Healthy tissue	ABL	26,8407	26,8407
46	Healthy tissue	CA9	32,5763	32,5763
46	Healthy tissue	CA9	32,9295	32,9295
47	Healthy tissue	ABL	27,9814	27,9814
47	Healthy tissue	ABL	27,9867	27,9867
47	Healthy tissue	CA9	33,0001	33,0001
47	Healthy tissue	CA9	32,9585	32,9585
48	Leukoplakia tissue	ABL	31,8639	31,8639
**Patient**	**Sample Group**	**Gene**	**Ct**	**Ct adjusted**
48	Leukoplakia tissue	ABL	31,8152	31,8152
48	Leukoplakia tissue	CA9	32,5078	32,5078
48	Leukoplakia tissue	CA9	32,913	32,913

49	Leukoplakia tissue	ABL	27,4748	27,4748
49	Leukoplakia tissue	ABL	27,4907	27,4907
49	Leukoplakia tissue	CA9	31,3805	31,3805
49	Leukoplakia tissue	CA9	31,1685	31,1685
50	Leukoplakia tissue	ABL	26,2855	26,2855
50	Leukoplakia tissue	ABL	26,4528	26,4528
50	Leukoplakia tissue	CA9	29,7789	29,7789
50	Leukoplakia tissue	CA9	29,681	29,681
51	Tumoral tissue	ABL	29,0815	29,0815
51	Tumoral tissue	ABL	29,2647	29,2647
51	Tumoral tissue	ABL	27,3958	27,3958
51	Tumoral tissue	ABL	27,4342	27,4342
51	Tumoral tissue	CA9	23,8009	23,8009
51	Tumoral tissue	CA9	23,6395	23,6395
51	Tumoral tissue	CA9	23,792	23,792
51	Tumoral tissue	CA9	23,7307	23,7307
52	Tumoral tissue	ABL	27,3867	27,3867
52	Tumoral tissue	ABL	27,6689	27,6689
52	Tumoral tissue	CA9	30,9779	30,9779
52	Tumoral tissue	CA9	31,2993	31,2993
53	Tumoral tissue	ABL	26,1574	26,1574
53	Tumoral tissue	ABL	25,9676	25,9676
53	Tumoral tissue	CA9	31,5139	31,5139
53	Tumoral tissue	CA9	31,4922	31,4922
54	Tumoral tissue	ABL	26,571	26,571
54	Tumoral tissue	ABL	26,4728	26,4728
54	Tumoral tissue	CA9	27,3532	27,3532
54	Tumoral tissue	CA9	27,4235	27,4235
